# Universal structures of normal and pathological heart rate variability

**DOI:** 10.1038/srep21749

**Published:** 2016-02-25

**Authors:** Alfonso M. Gañán-Calvo, Juan Fajardo-López

**Affiliations:** 1 Depto. de Ingeniería Aeroespacial y Mecánica de Fluidos, Universidad de Sevilla, E-41092 Sevilla, Spain; 2 Intensive Care Unit, Clínica Santa Isabel, E-41018 Sevilla, Spain

## Abstract

The circulatory system of living organisms is an autonomous mechanical system softly tuned with the respiratory system, and both developed by evolution as a response to the complex oxygen demand patterns associated with motion. Circulatory health is rooted in adaptability, which entails an inherent variability. Here, we show that a generalized N-dimensional normalized graph representing heart rate variability reveals two universal arrhythmic patterns as specific signatures of health one reflects cardiac adaptability, and the other the cardiac-respiratory rate tuning. In addition, we identify at least three universal arrhythmic profiles whose presences raise in proportional detriment of the two healthy ones in pathological conditions (myocardial infarction; heart failure; and recovery from sudden death). The presence of the identified universal arrhythmic structures together with the position of the centre of mass of the heart rate variability graph provide a unique quantitative assessment of the health-pathology gradient.

The heart is the first organic autonomous volumetric pump developed by nature, which allowed the giant leap of mobility to living organisms. Contractility and beat rate are basic functions of the heart. Heart rate variability (HRV) represents a vital evolutionary degree of freedom of an autonomous organism with a circulatory system^1^, which provides immediate responsive adaptability to oxygen demands. Those demands may have nearly infinite variability profiles. However, for economy principles, nature responds to demands creating a limited number of *structures* or patterns instead of infinite solutions. Thus, questions such as to which extent could HRV cling to predetermined structures or patterns, whether such structures -if any- would reveal general modes of adaptability (health) and failure (pathology), would those structures appear in combination, what would be their relative generalized size and weight, would those structures be complementary or antagonistic, etc. are the objects of this work.

The complementary respiratory system of mobile organisms living in air is also a volumetric pump (the chest-lungs), with another characteristic time softly tuned to that of the heart. The soft link between heart beat and breath rates entails a specific HRV that was early identified^2^,^3^,^4^,^5^,^6^. Heart beat is also subject to other endogenous inputs like digestion^7^, age and gender^8^,^9^,^10^,^11^,^12^, biochemical conditions^13^,^14^,^15^, or psychic related issues^16^,^17^,^18^,^19^, whose characteristic associated time is normally decoupled from that of the autonomous heart beat. When such inputs or conditions reach to, or overcome external inputs (including circadian cycle^20^), the organism may exhibit pathological arrhythmias. However, the most life-threatening are those for which the cardiac condition is challenged, with characteristic times below that of breath rate^16^,^21^,^22^,^23^. We hypothesize that universal specific arrhythmic sequences (internal structure) may exist in the human species as a co-evolutionary product of the sympathetic-parasympathetic system, and that those sequences might be specific to healthy or pathologic cardiac conditions.

The need for non-invasive, precise and specific tools of diagnosis and prognosis is a primary driver in the advancement of Medicine. Compact graphical representations of physiological systems have provided an immense advantage for physicians in terms of detail and precision in diagnosis. Imaging of internal systems and tissues (e.g. echography, CT-scan, or NMR) has changed our lives. This is why in cardiac quantification (CQ), echocardiography (echoCG) has represented a major development. Nonetheless, electrocardiographic data series provide irreplaceable, complementary information not accessible to echoCG in many aspects like pathologic temporal behavior and patterns, ectopic beats, etc. An ambitious, non-trivial task would be to determine the optimal measures in a Holter record (HR) time series that can be reduced to a compact graphical representation (or graph), similar and complementary to echocardiography in clinical value. A number or preliminary considerations is necessary: in order to emphasize the appearance of generic patterns reflecting either normal or pathological features, and enhance specificity, that graph should reduce to a minimum (i) the individual distinctions in mass/size, gender, age, etc., (ii) long term influences, with times comparable or larger than breath rate, and (iii) both exogenous and endogenous influences other than those of cardiac or circulatory origin. That graph should keep the complete information provided by the beat sequence (for example, the sequence of all beat to beat intervals, or RR intervals) as to be decoded to reconstruct the original extended sequence. To the best of our knowledge, no such graph exists to date.

Among the different approaches to study HRV, including tools from dynamical systems and chaos analysis^24^,^25^,^26^,^27^,^28^, multiscale entropy analysis^29^, analysis in the frequency domain^30^,^31^,^32^,^33^, and Markov chains^34^,^35^,^36^, Poincaré maps of RR intervals provide useful compact graphs of a HR^37^,^38^,^39^,^40^,^41^,^42^. In this work, we postulate that the high stochastic heterogeneity exhibited in general by HRV is a signature of strong internal and presumably universal structures. Under this thesis, the most suitable tools for HRV would be a normalized time sequence analysis in contrast to global, stochastic tools, or Fourier transforms. Indeed, Fourier transform may definitely not be the right tool to analyze universal variability patterns, because it essentially analyzes the content of *fixed* time scales in the whole record, thus mixing up patterns of potentially the same nature for high and low beat rates in the same HR.

Poincaré plots (or return maps) of RR series provide a direct time sequence analysis to identify temporal patterns. However, the limited range provided by planar (2D) return maps^41^ hamper the full potential of this graph. In particular, a 2D return map is a planar projection where complex trajectories with multidimensional features (i.e. specific arrhythmic sequences) appear superposed and indistinguishable. Here we propose a rational generalization (*N*–dimensions) of return maps using a normalized variability in terms of a moving average of *N–*order. It addresses the above mentioned demands for an ideal representation of cardiac function in terms of compactness and completeness, and the normalization proposed reduces to a minimum the exogenous or accidental influences. This procedure can be applied to any component of the QRS complex, although in this work we focus on the analysis of the principal component, the RR series. In this work, we have explored the range from order *N* = 2 to *N* = 100, observing that global variability quantifiers like the one introduced in the following (that we call primary variability) consistently exhibits a minimum for *N* = 5 in the case of healthy individuals. This might be due to the fact that the average cardiac rate is approximately five times that of breath rate in our species, which induces a subharmonic of order five in the HRV, thus maximizing the global compensation when *N* = 5. If this hypothesis is correct, deviations from the normal condition should be optimally distinguished at this order.

Fortunately, *N* = 5 yields the most complete, graphically representable graph of all orders: we will show that five subsequent normalized differences with respect to a local five-point average in a data series (e.g. RR intervals) provide a *sequential* information vector in *four* dimensions that can be graphically represented in 3D+ color to yield a compact, clinically descriptive and valuable comparative tool. This graph allows the identification of universal sequence patterns whose density distribution and combinations may provide an immediate and complete information on the condition of a cardiac system. In particular, we have identified up to five ubiquitous sequences particularly visible in our proposed graph, but not exclusively present when *N* = 5: indeed, all of them can be found at larger orders and their general expressions for any *N–*order have been obtained. The universality of those sequences is verified in a series of HR databases with 133 records grouped into four basic conditions with distinctive features of: (i) normal sinus rhythm (NSR) at normal activity or at supine rest, watching the movie “Fantasia”; (ii) ischemic cardiopathy (myocardial infarction, MI); (iii) heart failure (HF); and (iv) recovery from sudden death (SD). We show that the percentage of appearance of each sequence, and a properly defined primary variability provide an univocal and specific set of measures of physiological cardiac condition for those cases in the publicly available databases explored.

A HR is a set of *M* consecutive values corresponding to the RR intervals measured, that can be expressed as {*X*_*i*_}_*i*=1, ..., *M*_. We propose that the optimal way to study the general variability, independently of the particular state of the heart rate, is the use of the *generalized* distance from a particular point to the *identity line* (line of zero variability^43^), that distance made non-dimensional with the average heart rate 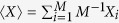 (see suppl. info). The identity line can be defined as {1, ..., 1}*t*, where *t* is any real positive number. Healthy hearts *dance* approximately around that line, but never sit on it (even in the extreme cases of more relaxed or more extenuating situations with apparent constant RRs, there is a mathematically non-zero variability). One can easily verify that the expression of the *k-* component of the local normalized vector distance from any normalized *i–*point 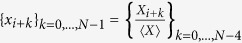 to the identity line {1, …, 1}*t* is simply given by:


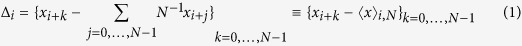


with *i* = 1, …, *M* − *N* + 1. In addition, only *N* − 1 of the *N* components of vector Δ_*i*_ would provide relevant information, since the extra component gives a redundant information once the other *N* − 1 components are known. Obviously, this is because the vector distance always belongs to the plane whose normal vector is {1, ..., 1}/*N*^1/2^. In other words, the vector Δ_*i*_ always sits on a (*N* − 1)-dimensional subset (a fixed plane), and thus its true informative dimension is *N* − 1. Besides, one can also define a *locally normalized* vector distance to the identity line as:


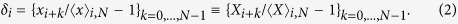


with 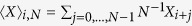.

Figure 1 shows examples of all this with *N* = 4, using a simple color code (0–1 hue code) as the fourth dimension. Both Δ_*i*_ and *δ*_*i*_ provide compact representations of the HRV [see Fig. 1(a,b)], with certain differences (other than the obvious): while Δ_*i*_ represents globally normalized variability, thus reflecting the actual, yet normalized extension of variability, *δ*_*i*_ is a measure of local variability everywhere along the record. Although the appearance of both graphs differ only slightly in reality (see Fig. 1(a,b)), one has that Δ_*i*_ often superposes certain features that appear as continuous regions [Fig. 1(a)], while *δ*_*i*_ exhibits their internal structure better [Fig. 1(b)]. Furthermore, *δ*_*i*_ results in a more compact [Fig. 1(b)], limited representation of variability that reflect universal features more clearly than Δ_*i*_. In many cases, this leads to the identification of different discrete modes of the same variability form, with different locally normalized amplitudes depending on the local average beat rate [Fig. 1(b)]. However, in this work we fundamentally use Δ_*i*_ to show the true universality of basic cardiac patterns in the human species, leaving the nuances of *δ*_*i*_ to further studies. Besides, the appearance of specific structures (streaks or lines) that appear in both Δ_*i*_ and *δ*_*i*_ is anticipated in Fig. 1.

Figure 2 shows the graph of three HRs according to the proposed procedure, for *N* = 5: Fig. 2(a) belongs to a normal healthy subject, and Fig. 2(b,c) belong to two patients with chronic HF. While the healthy subject show the same dull, compact feature around the origin as in Fig. 1(b), the subjects with HF show distinctive spatial lines which are subsequently visited following preferred sequences. Some immediate conclusions can be drawn. In first place, the lines exhibited are fundamentally straight. In some cases [e.g. Fig. 2(b)], a flat feature joins smoothly a space between two lines. There is a particular view angle (projection angle on the plane of view) that systematically reduces the main straight lines and planes to just three in all these cases [see Fig. 2(c)]. Using this view angle, we give the projections of all HRs in the databases cited in Material and Methods (NSR, NRS-Fantasia, MI, HF, and SD) in the supplementary information.

As it can be expected, the normalized Poincaré sections of the variability of the NSR database (suppl. info.) are relatively centered and homogeneously distributed around zero, showing a mathematical compensation within (apparently) a random series, i.e. a roughly spherical core with random (approximately Gaussian) distribution. A more detailed observation reveals that all cases show in reality a clear structure in the fourth dimension (color) along a specific direction. This direction, interestingly, is exactly the same for all individuals, independently of the shape of the distribution of points. This fact points to the existence of a subharmonic, probably related to the cycle of breath, reflecting an automatism of the sympathetic-parasympathetic system. This effect is comparatively rare or nearly absent in the individuals with MI, and cannot be appreciated in HF and SD. The existence of some individuals in the NSR database (about 12%) exhibiting features present in HF is subsequently quantified and discussed. The Fantasia database shows the same features (suppl. info.^44^,^45^) as those in the NSR database, and nearly the same percentage of subjects with features of HF. Besides, elder healthy subjects clearly show less variability than young people.

Nearly 70% of subjects of the HF database (suppl. info.^46^), and many of the SD studied (suppl. info.^47^) show the same distinctive lines previously mentioned. The percentage of subjects with homogeneously distributed clouds of points reverse as compared with NSR: less that 20%. On the other hand, the repetitiveness of patterns and their extension point to universal features of HRV. Finally, the SD database used in this study show patterns distinctively variable, and conspicuously more irregular than any other group. Some of them exhibit the same lines as those of HF, but most of them show a very complex or an apparently chaotic structure (see suppl. info.).

As a first feature of the graph here proposed (either in terms of Δ_*i*_ or *δ*_*i*_), one observes that it is centered around the origin by definition, being the density of points in different areas the specific signatures of variability. Thus, a primary measure of variability for a *N*th-order subharmonic and its global *compensation* (for example, in a 24-hour HR for a circadian framework) would be the norm of the normalized vector defining the center of mass of the graph made by Δ_*i*_. However, our experience shows that the whole record of subsequent vectors Δ_*i*_ for *i* = 1, ..., *M* − *N* produces an improper compensation due to the inherent nature of a Poincar’e plot: observe that the same arrhythmic RR interval *X*_*i*_ appears as a common component Δ_*i*−*k*,*k*_ in *N* − 1 vectors Δ_*i*−*k*_ (where Δ_*i*−*k*,*k*_ is the *k–*component of vector Δ_*i*−*k*_, with *k* = 0, ..., *N* − 2) around the identity line, which often produces a global apparent compensation. To avoid that, one may extract a subset of the whole series taking the index *i* in steps of *N*, i.e. *i* = {1, 1 + *N*, 1 + 2*N*, ...}), where each *X*_*i*_ appears just once. The distance from the center of mass of this subset to the origin, whose graph is nearly indistinguishable from that of the whole series except in their different densities, is given by:


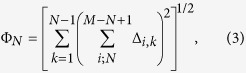


where Δ_*i*,*k*_ is the *k–*component of vector Δ_*i*_. This coefficient, that we term primary variability (PV), can be represented for each subject as a function of *N*. Our experience shows that Φ_*N*_ is about 10 to 100 larger than the distance to the origin of the center of mass of the complete original set, which drastically amplifies the significance of Φ_*N*_ as defined. It is also dependent on the number of beats in the series (see suppl. info.). In general, the minimum variabilities correspond to NRS (Fantasia), closely followed by MI. Besides, the maximum variabilities correspond to both SD and HF, being their distribution rather similar (see suppl. info.). Just in between, one has the NSR under normal activity. PV will be shown as a new quantitative measure with extraordinary distinctive values among cardiac conditions in combination with the graph here proposed and the arrhythmic structures subsequently deciphered.

In the following we provide a first classification of distinguished patterns that can be grouped into universal sequences. Moreover, we will show that the density of points along those lines provides a definitely valuable measure of cardiac condition. Indeed, when applied to the databases publicly available here used, our procedure yields a highly descriptive specificity for each group. As a first approach, in this work we limit ourselves to the identification of the most simple primary arrhythmic sequences that can be expressed in the form of a line as


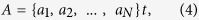


parametrized by an arbitrary variable *t*. Of course, the vector position of an actual point (beat) sitting approximately on an identified arrhythmic line may have any positive real *t* value. This reflects the ultimate essence of our idea: an arrhythmic line (or anomaly) in our normalized graph, which can be mathematically and universally expressed, gathers all arrhythmia *of the same nature*, independently of beat rate and variability strength. To calculate the density of points along the lines (sequences) present, several primary sequences have been deciphered and will be described in the following. These findings do not exclude the existence of other more complex sequences, with specific features associated, that can be sought for in future works.

One immediate way to assess the density of points corresponding to a specific sequence is to quantify its *presence* (in %) along the recorded RR series, as explained in Materials and Methods (see Numerical Methods). Given that the presence of a certain sequence is obviously not exact neither uniform amongst conditions and individuals, one needs to use statistical means. To do that, a convenient way to represent the *distribution* of the presence of a sequence under a given condition is plotting the value of *F*_*i*_ = *i*/*M* versus *y*_*i*_ for the corresponding database. Here, *y*_*i*_ is the percentage of presence of sequence *A*_1_, *i* is the rank of a particular subject based on his/her score *y*, and *M* is the total number of RRs recorded. This will be given for each sequence, and analyzed in combination for each condition.

*Arrhytmia A1.-* A normal subject with NSR should exhibit an intrinsic capability to respond to any demand of the organism by regular accelerations or decelerations of the heart beat rate promoted by the sympathetic/parasympathetic system. This capability should be reflected in the appearance of the simplest form of HRV, that can be expressed as a linear ramp:


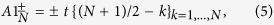


where the positive (+) or minus (−) sign apply for an accelerating or decelerating beat rate, respectively. For example, for *N* = 4 under accelerating beat rate, one has 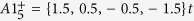; for *N* = 5 and decelerating beat rate, 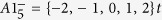; etc., where larger *t* values indicate a steeper ramp up of the heart beat, without any change in the functional structure of this variability.

This is the dominant form of HRV in a normal subject, as shown by its average presence (see Table 1) in the corresponding database (MIT BIH, NSR database). In fact, it is reflected by a slightly ellipsoidal shape around the origin in the direction of the line *A*1_5_ of any 4-dimensional graph of the fifth-order Poincaré map of HRV of a normal subject with NSR, as here proposed [see Fig. 2(a)].

More importantly, when the presence of this form of HRV decreases, other forms of pathologic arrhythmia subsequently described increase roughly in the same proportion. This fundamental finding points to a basic organic fact: this arrhythmia is in reality the basic *degree of freedom* of HRV reflecting the adaptability of a healthy organism. If any pathologic condition depresses or limits this degree of freedom, other forms of HRV raise to compensate that disability. Interestingly, these alternative forms are usually not arbitrary, particularly in conditions such as those classified as HF. Indeed, they are rather *universal*. Consequently, the specific form of these alternative HRV patterns should have ties to the specific condition that an organism undergoes, opening the door to new ways of non-invasive rapid diagnostics.

*Arrhythmia B1 (compensated ectopic beat).* The 4-dimensional HR graphs of subjects with HF usually exhibit three lines (Fig. 2(d)) that can be readily identified as (see Fig. 2(d)):


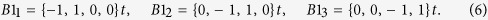


First, observe that these three sequences in reality belong to a single class of the type


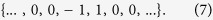


Second, the *average* value of the four components is zero for any *N* > 2, and therefore these sequences can be termed as *compensated*. This means that the last point of the Nth-order Poincaré section sits approximately on the identity line, i.e. Δ_*i*,*N*−1_ = 0. In other words, the sequence can be also termed as *terminated*, since the last beat interval lasts the same time as the local (moving) average. Interestingly, the number of beats with nearly zero variability around the sequence {−1, 1} is in most cases larger than two (i.e., the line {1, 0, 0, −1} does not clearly appear). Thus, one may conclude that there is a specific sequence described by the following line:


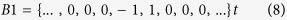


with a clear ubiquity. In the suppl. info. we show the consistency of the choice *N* = 5 as an optimum, convenient index to exhibit the presence of this sequence in a HR.

This type of arrhythmia is definitely characteristic and drastically dominant in HF condition, with a median of *y* close to one order of magnitude larger than in either SD or MI, and yet it is dominant in these cases as well. Interestingly, while the primary variabilities (PVs) of both HF and SD are large and very similar, what distinctively distinguishes HF from SD is a much larger presence of *B*1 in the former. These arrhythmias correspond to compensated, isolated ectopic beats; in this work, we do not attempt to link their specific cardiac origin or classification as premature ventricular complexes, atrial ectopic beats, etc. This analysis may provide a new ground for an overarching classification based on the inherent, strongly compensated nature of these arrhythmias and their reducibility to a single universally expressible structure. Besides, they are comparatively rare or absent in the NSR database. Consistently, ectopic beats can be shown in records of normal individuals, but are relatively rare. However, awaked subjects recorded at supine resting (Fantasia) exhibit more presence of this type of arrhythmias than normal subjects under normal activity. Notwithstanding this, the dominance of B1 in HF calls for a future revision on the diagnostic value^48^ (and references therein) of the presence of ectopic beats in combination with the capability to smoothly adapt (presence of linear ramps *A*1) to demands of normal activity. Indeed, one may observe in Table 1 the strong inverse correlation between the presence of compensated ectopic beats *B*1 and linear ramps *A*1 (±). The opposite combination of the presences of both *A*1 and *B*1 is clearly illustrated in Table 1, and particularly in Fig. 3.

*Arrhythmia B2 (regular paroxysmal tachycardia I)*. One may also graphically find the sequence described by [see Fig. 1(a,b)]:


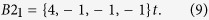


An additional line appearing in many cases [e. g. see Fig. 1(c,d)] is:


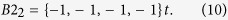


First, the appearance of these sequences introduces an additional source of randomization, combined with *A*_1_. This provides some extra adaptivity that would otherwise be severely limited (a stronger insufficiency would appear) as the presence of linear ramps *A*1 (normal adaptability) decreases. Secondly, neither one of those sequences is *compensated*. This means that one should consider higher order *N* values to seek for compensated or *terminated* sequences. In this case, both *B*2_1_ and *B*2_2_ are in reality part of the compensated sequence:


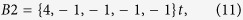


which in reality belongs to a sixth-order Poincaré section (i.e. *N* = 6). Interestingly, one finds that the compensated sequences described by lines of the type:


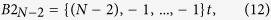


e.g.


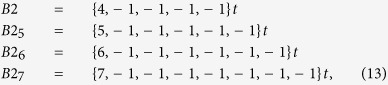


etc., have all nearly equivalent presences as *B*1 in HF, although they decrease as *N* increases. This sequence is hardly distinguishable as *N* increases above 10. In reality, the one with maximum presence is *B*2 (this is why we term this type of arrhythmia generically as *B*2 instead of *B*2_4_). An analysis of the presence of this arrhythmia in HF shows that it is as ubiquitous as *B*1, with different dominance ratios of one over the other, depending on the subject. This sequence is compatible with a regular paroxysmal tachycardia after a pause attributable to an atrioventricular blocking, in some cases leading to a Stokes-Adams syndrome (as the initial pause increases). Its average presence is given in Table 1 (and its distribution in suppl. info.). Again, like in the case of type 1, this arrhythmia type 2 is characteristic of HF. Notice that this type of arrhythmia represents a noticeable pause, followed by a proportional series of faster beats to compensate the pause.

*Arrhythmia B3 (regular paroxysmal tachycardia II).-* Several alternative sequences to *B*2 can be identified as:


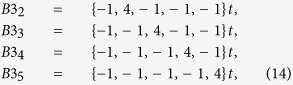


or


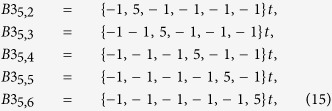


etc., that can be expressed for a general index *N* as:


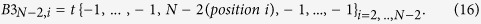


For a given *N*, all sequences *B*3 for different *i* indexes have similar presence, but significantly smaller than *B*2. Again, for *N* = 6, we simply drop the sub-index *N* − 2 = 4 since that is the most common form of this arrhythmia. It might be assimilated to intermediate situations joining two subsequent sequences corresponding to regular paroxysmal tachycardia with relative pauses as in *B*2. Interestingly, this arrhythmia is less characteristic of HF; in reality, it is as present in HF as it is in SD (with a nearly identical probability distribution). Moreover, it is significantly more present in NSR than in subjects with MI and in NSR (Fantasia).

A peculiar sequence appears in some cases as “shadows” of arrhythmias *B*2 in some cases [see for example Fig. 2(d)], which can be identified as the lines:


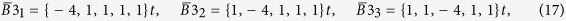


which can be written, in general, as


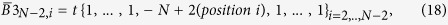


a sequence somehow complementary of *B*3_*N*−2,*i*_. Given the complexity of these types of arrhythmia, we are not providing a quantitative analysis of them.

*Arrhythmia A2 (breath-related).* A relatively ubiquitous sequence, yet having much smaller presence than *B*1 or *B*2 in HF or SD, appears as the sub-dominant form of arrhythmia in *normal* or asymptomatic subjects. That compensated sequence can be described by the following line:


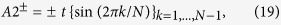


which represents a sinusoidal modulation of the beat rate along an interval of *N* beats. This type of arrhythmia with mathematical compensation should also reflect a physiological compensation. Given that it is statistically more common with *N* = 5 than with any other order, one may conclude its relationship with the breath rate. Whether it is more common during sleep than in normal activity is a subject of further investigations. Its presence is analyzed in Table 1 and in suppl. info. It is relatively dominant over the pathologic types in NRS. However, it is significantly less common in subjects with HF and SD. One may conclude that this arrhythmia is, like *A*1, characteristically *non-pathologic*. In other words, its presence is compatible with a *good cardiac health* condition.

The combination of densities of each identified arrhythmia may eventually constitute a valuable signature characteristic of a specific condition, which may open new ways to explore diagnostics means beyond the scope of this work. A summary of the results is provided in Table 1 (and in suppl. info.). Table 1 quantifies the average presence of the arrhythmias analyzed (except those of type 3) and the PVs in the different groups used in this study. A fundamental finding is the inverse relationship between the presence of certain types of arrhythmia that we term “healthy”, and others that we term “pathologic”. In particular, as far as the data in the public sources that we have used are reliable, arrhythmia *A*1^+^ and *B*1 are antagonistic: indeed, the relative presence of one over the other reverses as one considers normality or pathologic conditions. A discrete plot of a universal map where both presences are analyzed for NSR and HF is given in Fig. 3. Observe that the separation between NSR and HF provided by these two indexes is rather drastic.

In addition, PV values complete the set of characteristic variables that provide the most distinctive signature of each condition: observe in Table 1 that the combinations of the presences of each arrhythmia and the PVs configure a unique and strongly differentiated signature. Observe that what distinguishes MI from SD is precisely their respective PV, small for MI and large for SD. An approximate plot of the multivariate probability density functions for each condition in the space 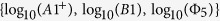 would be given by the density of points in that space corresponding to each condition. That is given in Fig. 3(b). The clinical value of this representation could be enormously significant. In fact, specific cardiotherapeutic drugs aimed at targets such as the sympathetic/parasympathetic system (beta-adrenergic-blocking drugs), or maybe the neuro-hormonal renin-angiotensin-aldosterone axis (ARBs, etc.), etc. may definitely displace the location of a certain subject in a predetermined direction that, to achieve therapeutic impact, should point to the NSR region. Finally, the potential prognostic value of results obtained from HRs applying our procedures becomes evident. The observation of the 4-dimensional graph of a HR here proposed allows the easy, immediate identification of anomalies in healthy people. A detailed analysis of some cases is given in suppl. info. Further clinical studies should expand in depth and width the analysis and results here briefly outlined, introducing new features and identifying new general arrhythmic patterns in relation with other pathologies and conditions not necessarily of a specific cardiac origin (e.g. diabetes, hyper- o hypothyroidism, etc., or even psychic conditions).

To conclude, we have proposed a quantitative procedure not only to provide a universal representation of the heart rate variability, but also a potentially powerful clinical tool to precisely qualify its cardiac condition, risks, and probably other related health issues. In this work, among many probably existing different universal sequence types, we have mathematically described some salient ones with relatively simple structure, identified as five general types of arrhythmia. The existing classifications of available databases according to cardiac conditions have allowed us to determine the signatures of NSR, MI, HF and SD as related to these basic types of arrhythmia. As a fundamental result, we have quantitatively shown that two of these arrhythmias are characteristic of *health*, while three are *pathologic*. Their relative presence in a subject may eventually be related to specific conditions as growing clinical evidence based on this methodology builds up in the future. Besides, the temporal evolution in a subject of the arrhythmic structures now visible through our methodology may provide invaluable information on his/her condition and/or clinical evolution. We believe that the potential of these results in simple, ambulatory and non-invasive clinical diagnosis and prognosis could be vital.

## Methods

### Local filtration and representation of variability

**Figure 1:**
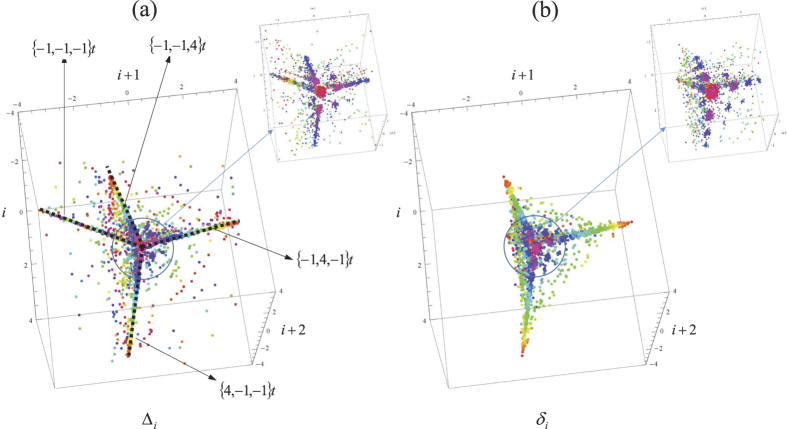
Representation of the distance vectors for a particular case of heart failure (**a**) {Δ_*i*_}, and (**b**) {*δ*_*i*_}. Some characteristic lines in this specific case and their mathematical expressions are shown. Also, zoom-in of the central regions of the graph are provided with smaller dot sizes to appreciate details.

An immediate way to achieve a compact representation of a complete HR series with a total number *M* of RR intervals, i.e. {*X*_*i*_}_*i*,1,..., *M*_, is to consider each element *X*_*i*_ (RR interval) as a point in a *N*–dimensional Poincaré map. Taking *N* = 4, a graphical representation of the *M* − 4 sequential sets of 4 consecutive RR intervals [{*X*_*i*+*k*_}_*k*=0, ..., 4_]_*i*=1, ..., *M*−4_ in the HR would serve that purpose (see Fig. 1a). However, this representation is utterly dependent on each subject, being potential universal features impossible to decipher. In order to investigate those potential features, we propose two levels of filtration as follows.

The method here proposed is based on a systematic scanning of the HR series computing the Nth-order forward moving averages. This procedure generates, from the original one, a number of alternative series with *M* − *N* + 1 elements. We propose the following algorithm:

1. Normalize the complete RR set in the HR with its average (circadian filtration level), i.e.


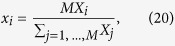


where *i* = 1, ..., *M*.

2. Calculate the normalized Nth-order forward moving averages (*N*–th order, local filtration level):


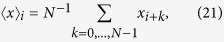


where *i* = 1, ..., *M* − *N* + 1, and 〈*x*〉_*i*_ is the value of the local *N*th-order forward average at the position *i* of the original HR series.

3. The plane defined by the equations


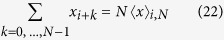


in a *N*-dimensional space is a *N* − 1-dimensional subset of that space. Then, the vector distance from the normalized point {*x*_*i*,*k*_} ≡ {*x*_*i*+*k*_} to the identity line is given by:


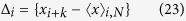


for *k* = 0, ..., *N* − 1 and *i* = 1, ..., *M* − *N* + 1. Alternatively, one can also define the locally averaged vector of distances as:


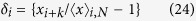


The simple algebraic equation (22) can be written for convenience in terms of the vectors of differences Δ_*i*,*k*_ or *δ*_*i*,*k*_ simply as:


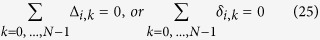


where Δ_*i*,*k*_ and *δ*_*i*,*k*_ are the components of vectors Δ_*i*_ and *δ*_*i*_, respectively, and *i* = 1, ..., *M* − *N* + 1. Obviously, given the first *N* − 1 components of vector Δ_*i*_. i.e. for *k* = 0, ..., *N* − 2, the last component Δ_*i*,*N*−1_ is known from equation (25). Thus, one can form a set of *M* − *N* + 1 vectors {Δ_*i*,*k*_}_*k* = 0, ...*N*−2_ truncated by the last component Δ_*i*,*N*−1_ which are representable as dots in a (*N* − 1)-dimensional space. The same applies to *δ*_*i*_. This representation defines a specific multi-dimensional body which constitutes a compact codification of the complete original series. When *N* = 5, that body can be represented in a three-dimensional space + color range (a 4-dimensional space).

### Identification of sequences

To verify whether a given sequence belongs to a given standard sequence (e.g. *l*_1_, *l*_2_, etc.) in a generalized space of *N* dimensions, we introduce the generalized angle *θ* formed by any sequence Δ_*i*_ with the line defined by any of the identified arrhythmia *A*, whose cosine is:


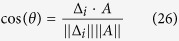


where 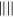 indicated the generalized norm of a (*N* − 1) dimensional vector. Thus, *θ* measures the deviation of any sequence from a given standard arrhythmia *A*. A way to quantify the presence of a given sequence along a HR is:

1. Measure *θ* around each beat of the series,
2. Count the number of times *m* that *θ* is below a certain tolerance 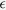.
3. Multiply previous number by (*N* − 1) and divide by the total number of beats in the sequence minus *N*. The coefficient 100 × *m*(*N* − 1)/(*M* − *N*) is the percentage of presence of the sequence *A* in the series.

### Experimental databases used

Holter records (HR) analyzed in this paper have been obtained from Physionet.org^49^. We have used the following Databases:

- Non-pathologic, normal activity records (normal sinus rhythm, NSR): MIT-BIH Normal Sinus Rhythm Database. This database includes 18 long-term ECG recordings of subjects referred to the Arrhythmia Laboratory at Boston’s Beth Israel Deaconess Medical Center. Subjects included in this database were found to have had no significant arrhythmias and include 5 men, aged 26 to 45, and 13 women, aged 20 to 50.
- Non-pathologic, awaked at rest (under homogeneous visual input): We studied Fantasia Database^44^,^45^, composed for twenty young (21–34 years old) and twenty elderly (68–85 years old) rigorously- healthy subjects in sinus rhythm, each subgroup including equal numbers of men and women. All subjects underwent 120 minutes of continuous supine resting watching the movie Fantasia (Disney, 1940) to help maintain wakefulness, while continuous ECG is recorded.
- Ischemic Cardiopathy (MI): European ST-T Database^47^. This is a project which goal was to prototype an ECG database for assessing the quality of ambulatory ECG monitoring systems. Thirteen research groups from eight countries provided 90 ECG records. From these records, we have selected those exhibiting MI, 29 cases in total.
- Heart Failure (HF): HRs were obtained from Congestive Heart Failure RR Interval Database^46^. This database includes the RR intervals for 29 patients aged 34 to 79, diagnosed with congestive heart failure NYHA classes I, II, and III.
- Sudden Death (SD): HRs, as used in^50^, were obtained from Physionet^49^.

## Additional Information

**How to cite this article**: Gañán-Calvo, A. M. and Fajardo-López, J. Universal structures of normal and pathological heart rate variability. *Sci. Rep.* **6**, 21749; doi: 10.1038/srep21749 (2016).

## Supplementary Material

Supplementary Information

## Figures and Tables

**Figure 2:**
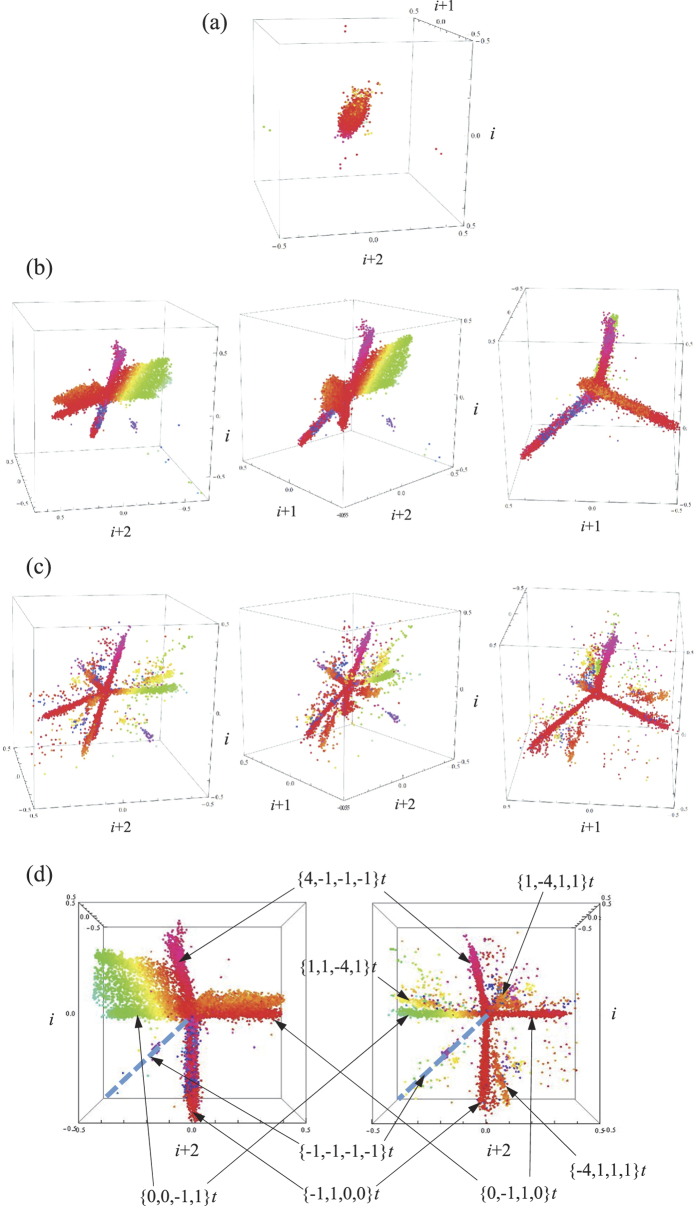
(**a**) 4-dimensional plot of the locations of the *M* − 3 vectors Δ_*i*_ (*i* = 1, ..., *M* − 3) of a normal HR (a healthy adult). (**b**,**c**) Same for two subjects with chronic heart failure (three view angles each). The plots in (**b**,**c**) at the right show the universality of patterns when projected with the appropriate angle. (**d**) Identification of the main lines in heart failure in two different subjects with HF.

**Figure 3:**
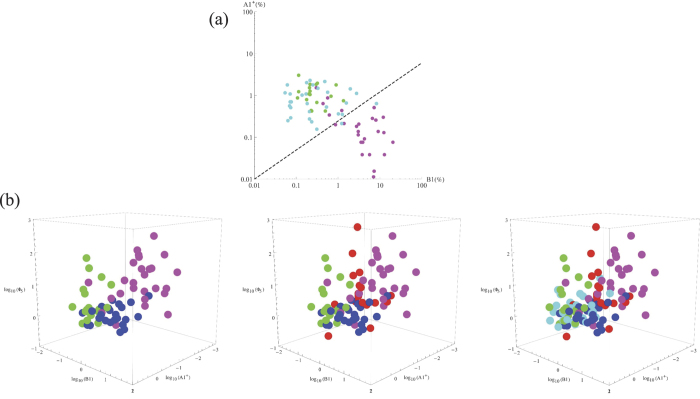
(**a**) The different regions of normal and HF conditions found in this study: NSR (normal activity -n.a.-, green; Fantasia, cyan); HF (magenta). (**b**) The different regions occupied by the four conditions found in this study in the multivariate space 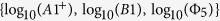: NSR (n.a., green; Fantasia, cyan); MI (blue); SD (red); HF (magenta). For better clarity, left panel provides three conditions (NSR n. a., MI and HF), the central panel four (NSR n. a., MI, SD and HF), and the right panel all conditions studied. *N* = 5.

**Table 1:** Average presence (in %) of the different arrhythmia types found in the databases.

Arrhythmiatype	NSR (normalactivity)	Fantasia	MI	SD	HF
A1^+^	1.21849	0.841977	0.682145	0.355535	0.23905
A1^−^	0.725413	0.657101	0.502548	0.264764	0.188382
A2^+^	0.417186	0.576313	0.320071	0.33573	0.158211
A2^−^	0.630867	0.58272	0.379129	0.284021	0.175146
B1	0.354003	0.568972	1.7985	1.48564	5.59225
B2	0.431622	0.32933	0.339216	0.493797	0.772125
Φ_5_	10.1739	2.4295	1.7567	41.2053	48.9619

Also given, the average primary variability Φ (*M* = 4200) for each database. We have used *N* = 5 and tolerance 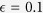 for all calculations in this Table.
