# Supplementary material for: Universal structures of normal and pathological heart rate variability
Source: Sci Rep. 2016 Feb 25;6:21749. doi: 10.1038/srep21749 (PMC4766475; doi:10.1038/srep21749)
Supplement: Supplementary Information [file srep21749-s1.pdf]

Universal structures of normal and pathological heart rate variability (Supplemental Information)

Alfonso M. Gañán-Calvo[1], Juan Fajardo-López[2]

[1]Depto. de Ingeniería Aeroespacial y Mecánica de Fluidos, Universidad de Sevilla, E-41092 Sevilla, Spain
[2]Intensive Care Unit, Clínica Santa Isabel, E-41018 Sevilla, Spain

January 14, 2016

A Holter record (HR) is generally a circadian record from which a set ξ of M consecutive RR intervals $\xi = \{X_i\}_{i=1,...,M}$ can be extracted, where X is the particular value of a RR interval in seconds. This set has a circadian average value $\langle X \rangle = M^{-1} \sum_{j=1,...,M} X_j$. Along the set, the patient heart beat is subject to alterations with respect to that average value. On the basis of demands, a normal heart modifies its beat rate following an adaptive path where a small variation compared to previous RR is accumulated along a given time interval. Normally, that interval comprise a relatively large number of beats whose length is dictated by the intensity of the demand and the physiological capabilities of the patient. If the variations of the RRs would be very small or zero, an $N-$dimensional projection of the set ξ involving the locations of the $M - N + 1$ vectors $\{X_{i+k}\}_{k=0,...,N-1}$ ($i = 1, ..., M - N + 1$) would approximately occupy the straight line, or (*identity line*, see for example [1]), defined as $\{1, ..., 1\}t$, where t is any real positive number. The mathematical justification for the existence of the identity

line and the natural variability around it can be relatively formally given as follows: the ideal continuous line (identity line) would be a solution to a dynamical system where the time required for adaptation, and the size of the sample, would both tend to infinity as the variability allowed is reduced to zero. However, a discrete set of points of a real system would never sit exactly on that line, unless the set is reduced to a series of identical RR values.

In the following, to illustrate the above we provide an example of a representation of variability by a direct 4-dimensional Poincar'e return map, using a color code (0-1 hue code) as the fourth dimension. Here, we plot the 4-dimensional vectors corresponding to four subsequent values of the RRs in seconds, taken along the entire series. This is *not* the procedure proposed in this work; we show this to illustrate a prior step to our generalized, normalized scheme. In this example, a healthy adult is subject to a HR, showing an average circadian heart beat RR about 1 second, and a typical variability of 5% of the average RR interval (50 ms). The stick-like representation of the set of $M-3$ vectors $\{X_{i+k}\}_{k=0,...,3}$ $(i=1,...,M-3)$ is given in Figure 1(a), while the four-dimensional anisotropic (nearly spherical) Gaussian distribution of the $M-4$ variability vectors $\{X_{i+k}-X_{i+k-1}\}_{k=1,...,4}$ $(i=1,...,M-4)$ of the same HR is given in Figure 1(b). The projections of Figure 1 (say, a *standard* Poincaré map) would help to perceive the particular appearance of each patient's output, but would hardly allow to extract quantitative universal features, given their particular (dimensional) nature and their dependency, among other conditions and inputs, on the average beat rate of a patient.

4-dimensional representations of the normalized variability vector $\Delta_{i,k}$

The four-dimensional graphs corresponding to the proposed representation of HRs in the databases analyzed in the main text are here provided in a case-to-case basis.. It should be said that specific features exhibited below

like the three main lines appearing in most HRs of HF (and less commonly in NSR, and NSR-Fantasia) may not be completely different sequences in reality, but *nearly* the same one where the pointer $i-$ is subsequently displaced.

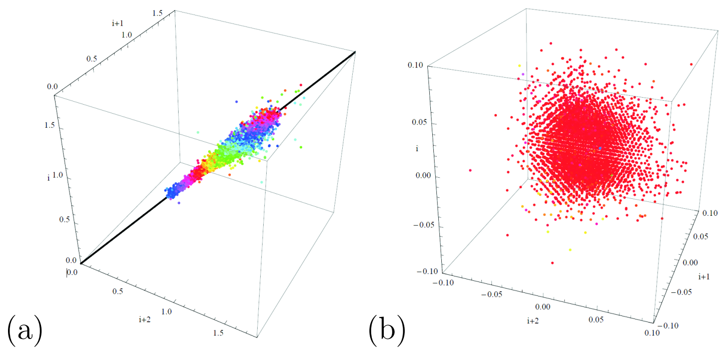

Figure 1: (a) 4-dimensional plot ($N = 5$) of the locations of the $M-3$ vectors $\{X_{i+k}\}_{k=0,\ldots,3}$ ($i = 1, \ldots, M-3$) of a normal HR (a healthy adult). The identity line (theoretical location of a quasi-perfectly regular heart beat) is marked as a black straight line. The fourth dimension is the color code defined as the hue (0-1) (b) 4-dimensional plot of the locations of the $M-4$ variability vectors $\{X_{i+k} - X_{i+k-1}\}_{k=1,\ldots,4}$ ($i = 1, \ldots, M-4$) of the same normal HR. Observe that the typical variability is around 5% of the average RR value, around 1 second.

The obvious differences among them are the previous and subsequent values of normalized differences, as the pointer $i-$ changes.

MIT-BIH NSR database

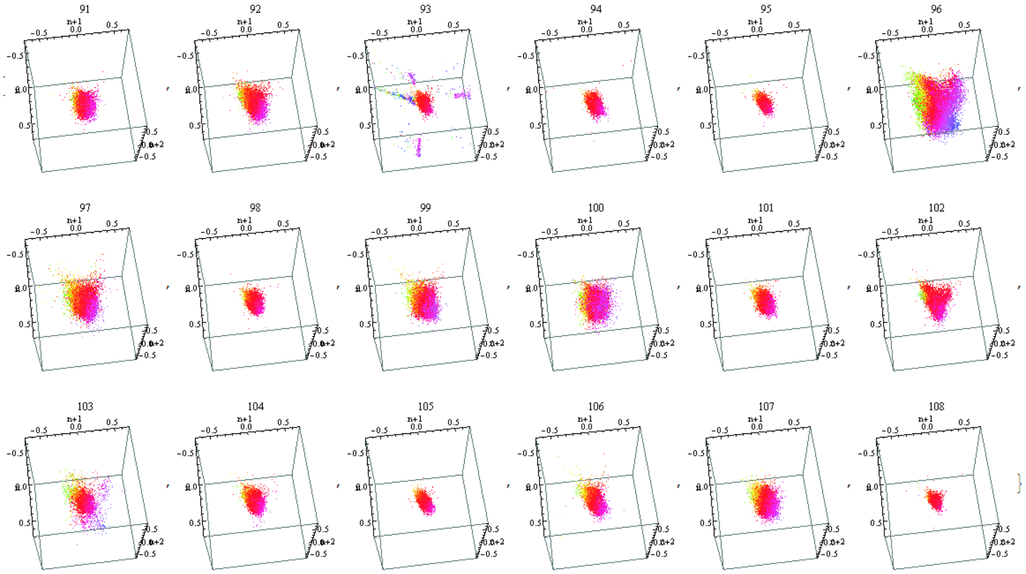

Figure 2: The 18 cases from the MIT-BIH Normal Sinus Rhythm Database ($N = 5$).

Fantasia database

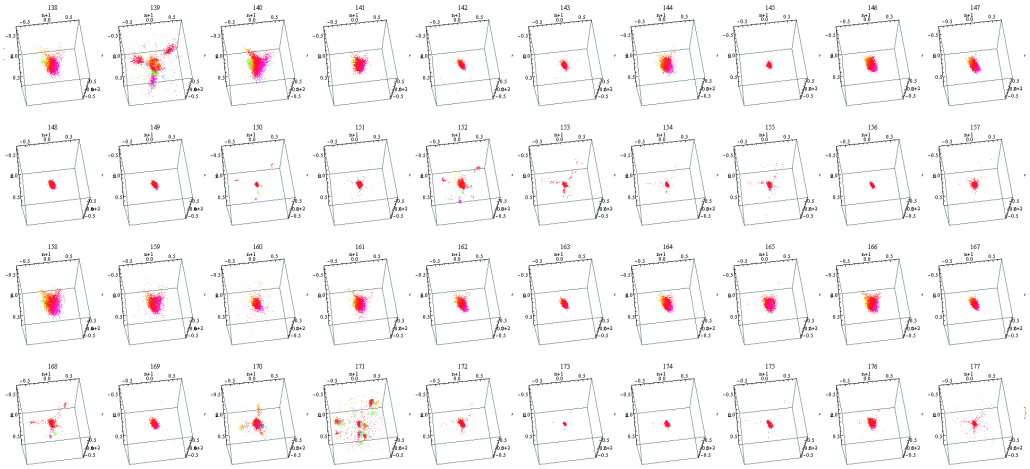

Figure 3: The 40 cases of the Fantasia Database ($N = 5$).

Ischemic Cardiopathy (MI)

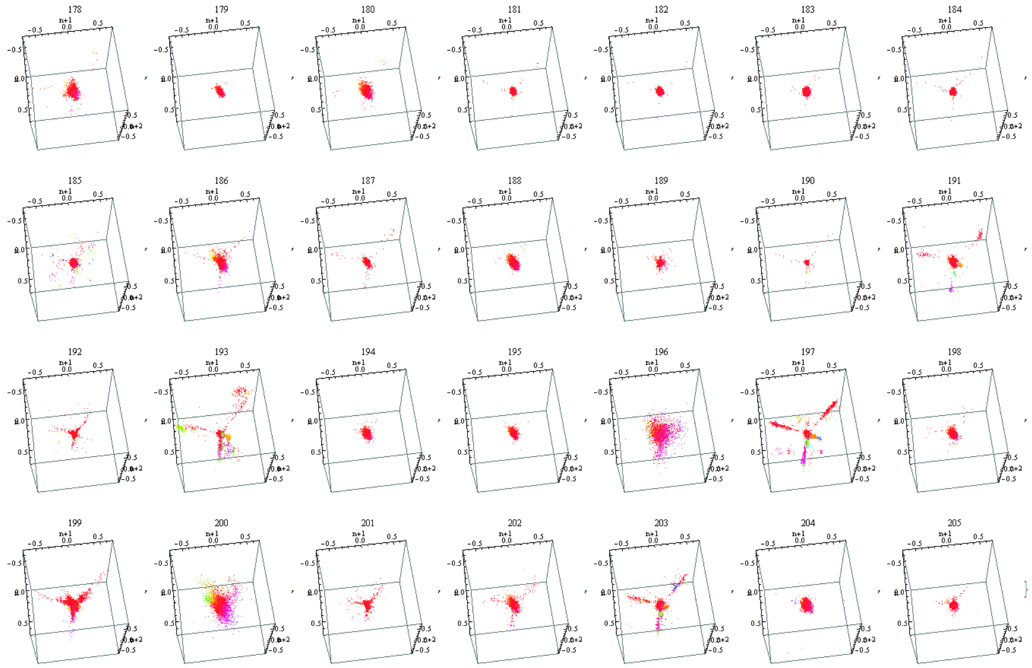

Figure 4: The 29 cases of Ischemic Cardiopathy showing myocardial infarction (MI) from the Ischemic Cardiopathy: European ST-T Database ($N = 5$).

Congestive Heart Failure (HF) RR Interval Database

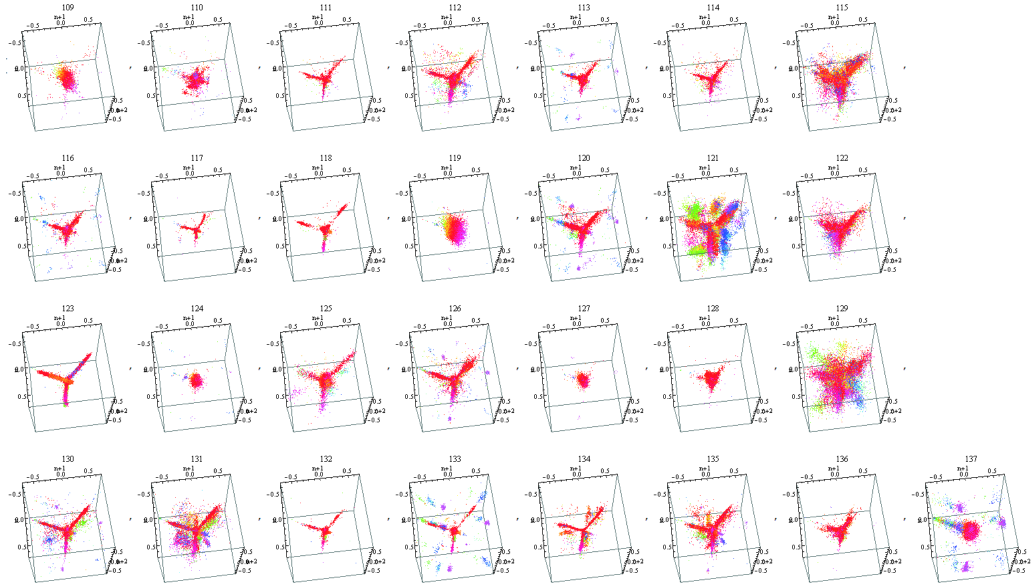

Figure 5: The 29 cases of heart failure from the Congestive Heart Failure RR Interval Database ($N = 5$).

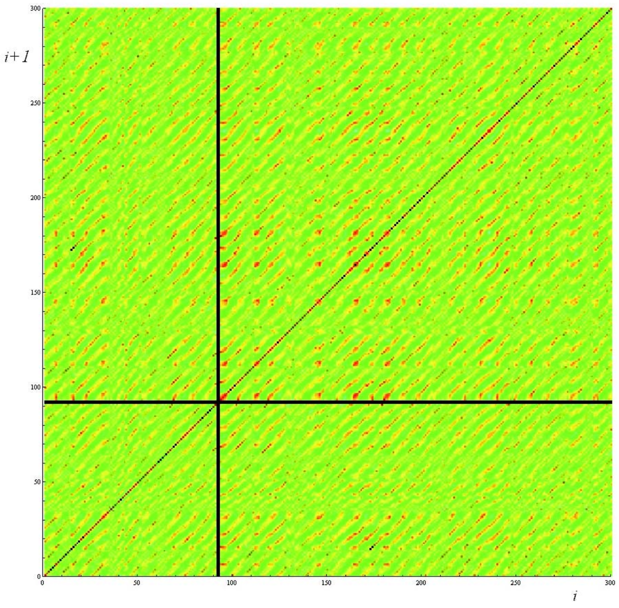

Figure 6: Autocorrelation map of 300× correlative vectors $\{\Delta_{i,k}\}_{k=1,\ldots,4}$ ($N = 5$) in the HR of the subject of Figure 3(b). The autocorrelation is measured in terms of the value of $\Theta = \arccos\left(\frac{\{\Delta_{i,k}\}\cdot\{\Delta_{i+1,k}\}}{\|\{\Delta_{i,k}\}\|\|\{\Delta_{i+1,k}\}\|}\right)$.

A planar autocorrelation map of a sequential subset of vectors from the whole HR of case "123" for $N = 5$ is shown in Figure 6. This map shows distinctive strips marking the propagation of the sequence $..,0,-1,1,0,...$ along -at least- six strokes in the ample majority of cases. The sequence appears, as marked by the horizontal or vertical separation among the strips, with a statistically dominant length of 5 (matching the choice $N = 5$ used to originate the map; other choices provide mismatching lengthes).

Recovery from Sudden Death (SD)

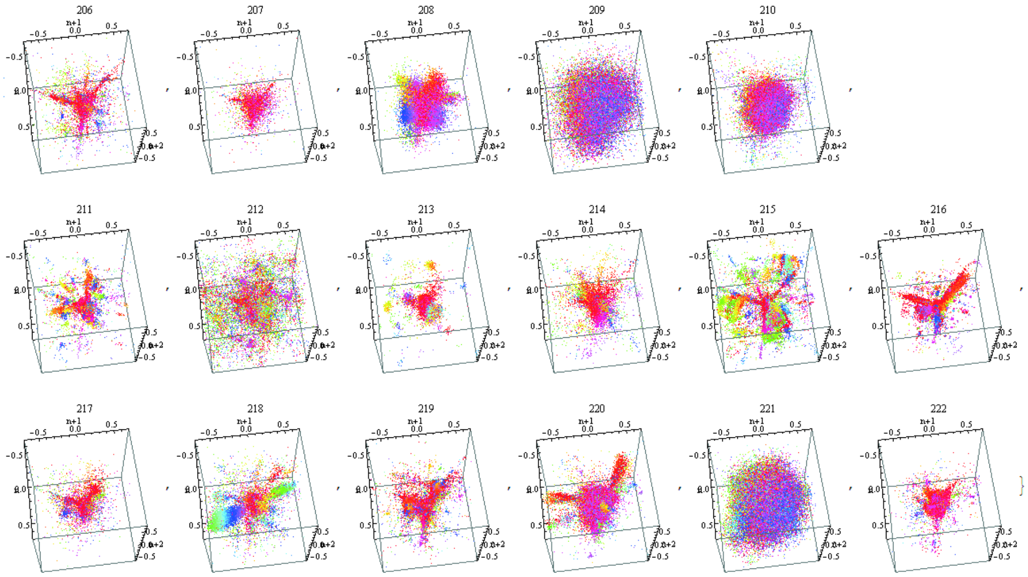

Figure 7: The 17 cases of recovery from Sudden Death studied ($N = 5$).

Primary Variability Φ_N

Primary variability (PV) is a first global measure from the graph obtained applying our procedure on a HR. It measures the global displacement of the *center of mass* of the graph from the origin, the *ideal* or theoretical center of mass of a perfectly compensated random sequence series. In other words, the theoretical mean of a multivariate (e.g. Gaussian) probability density distribution centered on the origin would be the null vector. PV reflects the inherent temporal nature of the HRV. Again, even if a subject exhibits a non-zero displacement of her graph from the origin, if her HRV would be statistically invariant with time, then that displacement would be constant independently of the number of beats of the HR series, for large M values (e.g., a circadian HR). However, that is not the case in all real HRs analyzed. Intriguingly, that dependency results in average as $\Phi_N \sim M^{0.2}$ for $M >> 1$. The complexity of this issue, involving a multiplicity of entangled dependencies with a unique outcome, is well beyond the scope of this initial work.

We have calculated the average value of this coefficient for 18 healthy subjects with NSR at normal activity (MIT-BIH Normal Sinus Rhythm Database), as a function of N. It is plotted in Figure 8(a). Φ_N asymptotically decays to zero approximately as $N^{-1/2}$ for $N \to \infty$. Nonetheless, the existence of local minima is evident, the most conspicuous one at $N = 5$ as expected. That would be expected given that the average hear beat rate is about five times that of breath, and the variations associated to breath are statistically filtered. Other sub-harmonics can be identified along the plot. This calculation may resemble a representation of the HR series in the frequency domain. However, Fourier transform is definitely not the right tool to analyze universal variability patterns, because it fundamentally uses *fixed* time scales along the whole record, thus mixing up patterns of the same nature for high and low beat rates in the same HR.

Figure 8(b) provides the approximate (discrete) cumulative distribution $F(\Phi_N)$ of primary variabilities for $N = 5$, i.e. Φ_5, for each condition: NSR (normal activity or Fantasia), MI, HF, and SD. The plot represents $F_i = i/M$ versus $\Phi_{N,i}$ for each subject of the corresponding database. Here, i is the rank of a particular subject based on his/her score Φ_N, and M is the total number of RRs recorded. F_i is the discrete approximation of the function of probability $F(y) = \int_0^y f(x)dx$, where $f(x)$ is the probability distribution function considered..

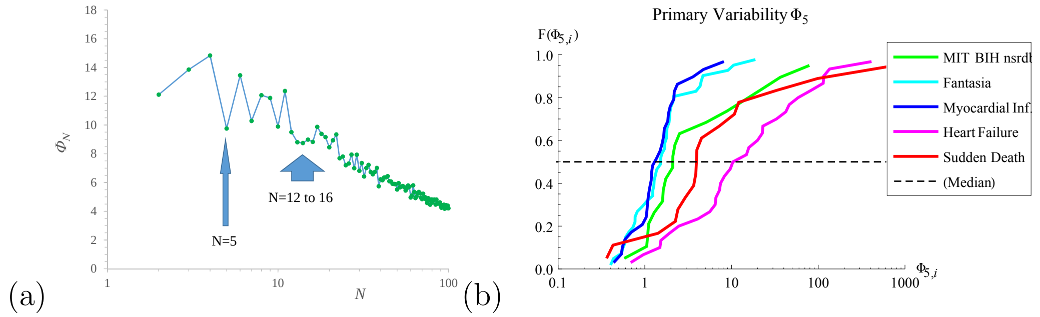

Figure 8: (a) Average values of the primary variabilities Φ_N of the subjects in the MIT-BIH Normal Sinus Rhythm Database, as a function of the index N. ($M = 65000$) (b) Approximate, discrete cumulative distribution of the primary variabilities (PVs) for each condition (NSR, normal activity or Fantasia, MI, HF, and SD), and $N = 5$ ($M = 4200$; this limitation is imposed by the length of the records in the "Fantasia" database).

Statistical distribution of presence of arrhythmias

Figure 9 represents the approximate (discrete) distribution of the presence of each arrhythmia classified by cardiac conditions.

One may observe in Figure 2 that the NSR (normal) cases "93", "96", and in some extent "102", present certain irregularities. Interestingly, their study reveals that cases "93" and "102" exhibit a deficiency of both $A0$ and $A4$ (healthy) in favor of either $A1$ or $A2$ (pathologic), and enhanced PV, see Table below. Both "93" and "96" cases point to characteristics of HF; case "96" exhibits mixed weights of healthy and pathologic arrhythmias, with a depressed PV contrary to what the graphic appearance of the HR might suggest. This shows that the arrhythmias are globally *compensated* and therefore no risk of HF would be present; however, the decompensation of $A0+$ and $A0^{-}$ might be compatible with possible issues in the sympathetic/parasympathetic system, or from a psychic origin.

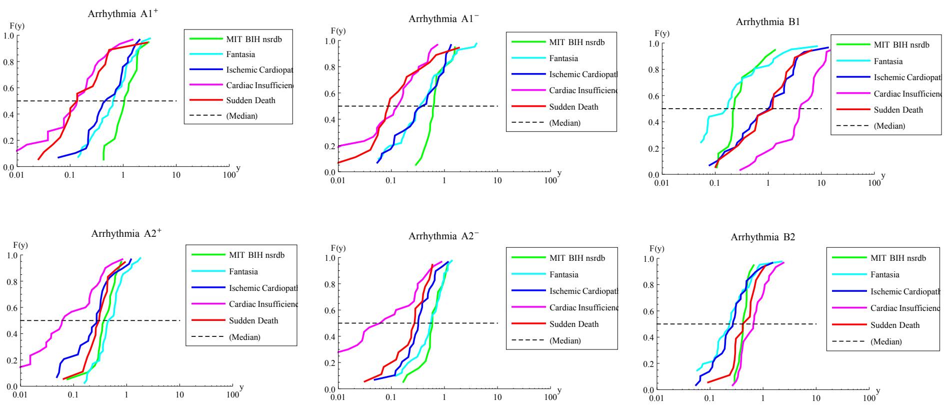

Figure 9: Discrete, approximate cumulative distribution functions of the presence of each type of arrhythmia in databases [NSR, normal activity or Fantasia, Ischemic Cardiopathy (MI), HF and SD]: Non-pathologic (Arrhythmias types A1 and A2), and pathologic (Arrhythmias types B1 and B2). $N = 5$, tolerance $\epsilon = 0.1$. The numerical precision of available records fail to provide reliable data below certain level, which provokes a cut-off in the value of the presence (rounded to zero) and is not represented in a logarithmic scale.

Database	NSR(*)	"93"	"96"	"102"
$A1^+$	1.227	1.03265	1.53374	0.96145
$A1^-$	0.767638	0.60429	0.534139	0.40442
$A2^+$	0.427822	0.344216	0.457834	0.289961
$A2^-$	0.637917	0.535447	0.923298	0.328114
B1	0.351007	0.214179	0.206025	0.68675
B2	0.416122	0.512499	0.663859	0.351006
Φ_5	8.12446	36.5542	1.74433	22.9644

Table 1: Presence (in %) of the different arrhythmia types found in some cases of NSR, as compared to the average values ($N = 5$, $\epsilon = 0.1$). (*) Excluding cases "93", "96", and "102".

References

[1] Piskorski, J. & Guzik, P. Compensatory properties of heart rate asymmetry. *Journal of Electrocardiology* **45**, 220–224 (2012).
